# Neural dynamics of visuospatial endogenous attention: Event-related optical signal evidence from posterior brain areas

**DOI:** 10.1162/IMAG.a.1176

**Published:** 2026-03-23

**Authors:** Giorgia Parisi, Sonia Mele, Elisabetta Colombari, Chiara Mazzi, Silvia Savazzi

**Affiliations:** Perception and Awareness (PandA) Laboratory, Department of Neuroscience, Biomedicine and Movement Sciences, University of Verona, Verona, Italy

**Keywords:** visuospatial attention, Posner paradigm, endogenous orienting, endogenous reorienting, contextual updating, optical imaging

## Abstract

Considerable research has been encouraged by the concept of two distinct human attention networks (i.e., a dorsal network, DAN, and a ventral network, VAN) for voluntary deployment of attention and the reorientation to unexpected events, respectively. Despite the general agreement about the main crucial nodes constituting the two networks, the specific contribution of each region and the interplay between the two systems required for flexible attentional control still need to be clarified. Therefore, the current study aimed to describe the neural dynamics of endogenous attentional processes and examine the predictive interactions between and within the two attention systems, by administering a detection and visuospatial version of the Posner paradigm and collecting data by using Fast Optical Imaging, coupled with Granger Analysis. Functional analyses uncovered bilateral dorso-parietal and visual recruitment during both orienting and reorienting. Importantly, a recursive predictive interplay between these dorso-parietal and visual regions was revealed to subserve both attentional processes. Additionally, a specific contribution of the left ventral network was found in allocating attention after the occurrence of a central predictive cue. In contrast, the right ventral network activity, which was actually elicited by the reorienting mechanism only, could indicate post-perceptual updating of the internal task-related mental models.

## 1 Introduction

Endogenous allocation of attentional resources in the spatial domain refers to the ability to prioritize and selectively attend to locations, including behaviorally relevant events. More specifically, during endogenous orienting, visuospatial attention is controlled by a goal-directed behavior and voluntarily focused on locations containing salient stimuli. Likewise, when behaviorally significant events occur in unexpected positions, visuospatial attention is reoriented toward them, establishing attentional reorienting. Several studies have explored the brain regions serving attentional orienting and reorienting (Corbetta & Shulman, 2002; Corbetta et al., 2000; Downar et al., 2001), proposing the existence of two distinguishable, although intertwined, frontoparietal cortical systems: a dorsal and a ventral frontoparietal network (DAN and VAN, respectively). These networks emerge to be both anatomically segregated and functionally dedicated to different processes of visuospatial attention (Vossel et al., 2014): DAN is bilateral and comprises the superior parietal lobule (SPL), the intraparietal sulcus (IPS), and the frontal eye fields (FEF). In contrast, VAN is deemed more right-lateralized and comprises the temporoparietal junction (TPJ) and the ventral frontal cortex (with particular reference to the middle frontal gyrus, MFG, and the inferior frontal gyrus, IFG) (Corbetta et al., 2008). From a functional standpoint, DAN is thought to generate and carry on the cued voluntary deployment of attention. In contrast, VAN is supposed to be responsible for reorienting visuospatial attention to unexpected behaviorally relevant locations (Corbetta & Shulman, 2002).

Despite their specialization, DAN and VAN do not operate in isolation but interact to render orienting and reorienting mechanisms efficient. Besides the anatomical linking between DAN and VAN, which is assumed to be subserved by different white matter tracts as the superior longitudinal fasciculus (Mengotti et al., 2020) (SLF) and the parietal inferior-to-superior tract (Catani et al., 2017) (PIST), a clear understanding of the contributions of both networks is still lacking. Transcranial magnetic stimulation (TMS) studies (Ahrens et al., 2019; Capotosto et al., 2012; Chica et al., 2011) have ascribed both endogenous orienting and exogenous reorienting to right IPS. In contrast, right TPJ has been found to be involved in attentional reorienting to unattended but task-relevant stimuli only.

Moreover, imaging studies revealed significant connectivity between right IPS and right TPJ during cued visuospatial tasks (Vossel et al., 2012; Wen et al., 2012). In addition to Vossel et al. (2009) highlighting right SPL activation in response to invalid cued targets in a location-cueing paradigm, Proskovec et al. (2018) observed functional involvement of bilateral SPL contrasting invalid against valid targets in a magnetoencephalography (MEG) study. Therefore, albeit different attentional subprocesses are selectively managed by either DAN or VAN, the literature supports the need for a flexible and dynamic interaction to accomplish an effective attentional performance.

Furthermore, attentional processes are not neurally implemented in frontoparietal and ventral regions only; instead, striate and extrastriate visual areas are implicated in these cognitive mechanisms as well (Chica et al., 2013). In line with this concept, previous research has shown the engagement of visual areas (i.e., cuneus) in anticipatory deployment of attention (Simpson et al., 2011) and the development of directed attentional effects from frontoparietal regions to visual areas during location-cueing paradigms (Bressler et al., 2008; Vossel et al., 2012).

Nevertheless, although there is evidence about the existence of an interaction between DAN and VAN along with the contribution of sensory regions during attentional tasks (Mengotti et al., 2020), more needs to be understood about the temporal dynamics of this interplay. To clarify the contribution of each node of these networks and how they predictively dialogue with each other constitute a still unanswered question, especially as regards the attentional reorienting process. For instance, right TPJ (rTPJ), constituting one of the cortical hubs of VAN, has been related to multiple cognitive functions, leading to a heated debate about its precise functional role in supporting attentional processes. At first, rTPJ was hypothesized to be involved in the reorienting mechanism by acting as an early circuit breaker which interrupts the ongoing deployment of attention carried out by the DAN and enables the latter to reorient attention toward unexpected but task-relevant stimuli (Corbetta & Shulman, 2002). Nonetheless, more recent and varied results have ruled out this theory, mainly ascribing to rTPJ a post-perceptual role by updating internal models of the attentional context to generate proper expectations and actions (Geng & Vossel, 2013).

This possibility is corroborated by the results of a previous work (Parisi et al., 2020) using fast optical imaging data. In this study, the authors unraveled functional relationships among cortical brain areas during endogenous orienting and reorienting, which was pointed out through a modified visuospatial version of the Posner paradigm (Posner, 1980). Participants performed a discrimination task where the cue direction was consistent throughout each block while the order of the blocks was alternated. Trials could be valid (75%) or invalid (25%). With regard to the reorienting mechanism, which was studied by analyzing invalid trials, the authors showed a later and recursive functional recruitment of rTPJ. They indeed suggested an early mutual interaction between visual and dorsal regions, which seems responsible for the different attentional sub-operations (i.e., encoding of the mismatch between expectation and reality, disengaging attention from the cued location, and triggering reorientation to the target) and communicates to the rTPJ only at later timeframes, accordingly ascribing to it a post-perceptual role in updating the preexisting internal model instead of triggering the reorienting process.

In the present study, we thus aimed at describing the neural spatiotemporal dynamics of visuospatial attentional processes by manipulating the paradigm employed in Parisi et al. (2020), seeking to return it as similar as possible to its classical visuospatial version proposed by Posner which constitutes an excellent behavioral model to investigate attentional orienting and reorienting and to further disentangle the latter from updating processes (Arjona Valladares et al., 2017; Käsbauer et al., 2020). To this aim, a simple detection task (instead of a discrimination task) was administered, and a random cue indicating toward either the left or the right hemifield was used instead of showing a consistent cue within a single block. By means of these manipulations, we intended to study attentional processes with the lowest cognitive demand to disengage the contribution of the posterior nodes of the DAN and VAN, with particular reference to cast light on right TPJ recruitment and to point out their order of neural activation and the predictive relationships among them.

Finally, functional data were collected by means of Event-Related Optical Signal (EROS) or Fast Optical Signal (FOS) (Chiarelli et al., 2013, 2014; Chiou et al., 2024; Gratton & Fabiani, 2001; Gratton et al., 1995; Perpetuini et al., 2023). Unlike traditional fNIRS, which captures the slower hemodynamic response following task execution, the EROS technique uses near-infrared light (NIR) to detect fast changes in light scattering directly linked to neuronal electrical activity. The reduction in light scattering is indeed associated with fluctuations of the membrane potential, allowing EROS to provide a measure of neuronal activity with a temporal resolution in the order of milliseconds (i.e., about 25 ms), which is comparable with what can be obtained with electroencephalography (EEG) and magnetoencephalography (MEG). Additionally, the arrangement of the optical probes constrains the path of NIR light across the brain, resulting in sufficiently spatially localized signals (on the order of centimeters). This provides a spatial localization power superior to EEG and MEG while revealing activity from extensive areas of the cortical surface (Gratton & Fabiani, 2010). Accordingly, EROS represents an optimal tool to obtain new insights into the time course of attentional processing in parallel with precise identification of cortical regions (Colombari et al., 2024; Kubota et al., 2020; X. Z. Xiao et al., 2022).

## 2 Methods

### 2.1 Participants

Thirty healthy volunteers were recruited for the study (eight males). Their ages ranged between 20 and 37 years (mean age ± standard deviation: 24.7 ± 3.4), and they were all right handed, as assessed with the Edinburgh Handedness Inventory (Oldfield, 1971). All reported normal or corrected-to-normal vision and no history of neurological or psychiatric disorders. All but one author (E.C.) were naïve to the purposes of the study. Written informed consent was obtained from all participants for being included in the study, which was approved by the ethics committee of the Verona Azienda Ospedaliera Universitaria Integrata (AOUI) and carried out according to the principles laid down by the 2013 Declaration of Helsinki. All participants received compensation for their participation.

Data from three participants were excluded from the analysis as being behavioral task outliers. Moreover, data from another participant were discarded because of digitization issues. Thus, the final sample comprised 26 participants (7 males, mean age ± standard deviation: 23.7 ± 2.4 years).

### 2.2 Experimental procedure

To prevent tiredness and achieve an acceptable number of trials, participants performed two distinct experimental sessions over 2 days. There were no differences between experimental sessions in setting and behavioral conditions, excluding EROS montages (see below). Each session lasted about three and a half hours and consisted of EROS setup, optical data recording during the behavioral task, and co-registration procedures (i.e., the digitization of optode scalp locations).

### 2.3 Behavioral task

Participants were individually tested in a dimly lit testing room. During the experiment, they sat in front of a 17-inch LCD monitor (resolution 1920 × 1080, refresh rate of 144 Hz) placed at a viewing distance of 57 cm with head position stabilized by an adaptable chin rest so that eyes could be adjusted to the center of the screen.

**Fig. 1.**
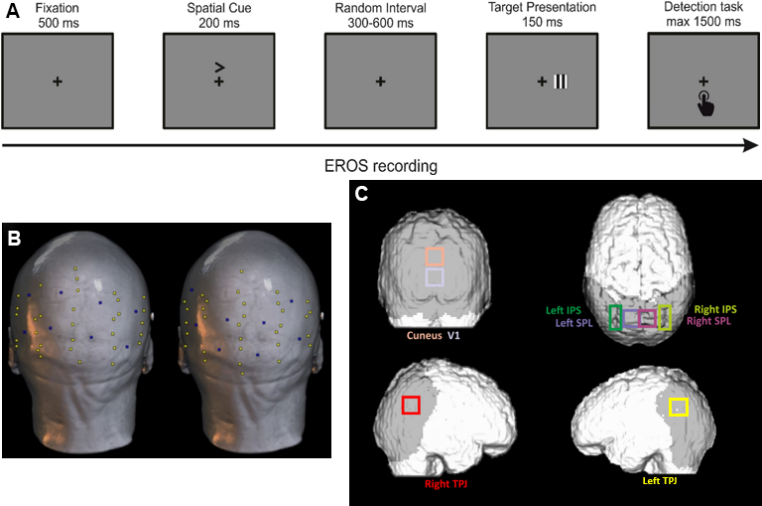
Method. (A) Experimental paradigm. A fixation cross was presented for 500 ms, followed by a central predictive cue lasting 200 ms. After a random interval ranging from 300 to 600 ms, the visual target occurred (for 150 ms), giving participants 1500 ms to respond to it. In this example, a valid trial is displayed (i.e., the cue indicates the same visual hemifield in which the target is subsequently presented). (B) Optical montages. Two recording montages were used for each helmet size. Infrared optical sources (yellow dots) and detectors (blue dots) were placed to cover the parietal and occipital cortices maximally. Here, source and detector locations are plotted on the anatomical scan of a representative participant. (C) Selected ROIs. Estimated boundaries of the selected ROIs used for EROS and GCA. ROIs are displayed in coronal (visual regions), axial (dorsal regions), and sagittal (ventral region) views. ROIs coordinates are listed in Table 1.

A cued detection task was administered (Posner, 1980). Stimuli were generated using E-Prime 2.0 software (E-Prime Psychology Software Tools Inc., Pittsburgh, PA, USA) and consisted of vertical or horizontal, black-and-white, 2° square gratings. Participants were instructed to maintain fixation on a centrally presented black cross, which 500 ms later was followed by a predictive random cue above the fixation cross (duration 200 ms). After a random interval, ranging from 300 to 600 ms, the target was presented for 150 ms at an eccentricity of 2° from the fixation cross to the inner edge along the horizontal meridian. Each stimulus of a single trial was displayed on a gray background (see Fig. 1A). Participants were to respond as fast as possible to the target by pressing the space bar of the keyboard with the index finger of their right hand in half of the blocks, alternating with the index finger of their left hand in the other half (the order of the hand was counterbalanced across both blocks and participants).

In each block, horizontal and vertical gratings randomly occurred with the same probability to avoid habituation. Moreover, trials could be valid (75%), that is, when the target appeared on the side indicated by the cue, or invalid (25%) when the target appeared on the opposite uncued side.

Each experimental session was composed of 24 blocks (for a total of 48 blocks per participant). Each block consisted of 48 valid trials, 16 invalid trials, and 16 catch trials (no target after the cue presentation) for a total of 3840 trials (2304 valid, 768 invalid, and 768 catch trials) per participant.

Participants could rest during inter-block intervals and initiate the next block by pressing a key.

### 2.4 Optical recording

Simultaneously with behavioral data acquisition, brain activity was recorded by means of two synchronized frequency domain oximeters (Imagent, ISS, Inc., Champaign, IL). Near-infrared light (830 nm, i.e., a wavelength chosen to optimize penetration depth, limit scattering, and maintain sensitivity to neuronal activity (Chiarelli et al., 2014)) was emitted by 32 laser diodes, modulated at 110 MHz. The light was directed to the participant’s head through 400 µm optic fibers and then detected by eight 3-mm fiber-optic bundles connected to photomultiplier tubes (PMTs). The detectors were modulated at a slightly different frequency in relation to laser diodes, generating a signal with a 3125 Hz cross-correlation frequency. PMTs’ output current was then processed by Fast Fourier Transform to obtain measures of the signal’s DC intensity, AC amplitude, and relative phase delay (source to detector). Only changes in phase delay data (converted into picoseconds delay) were examined in this study.

Custom-built helmets, available in two sizes, were used to secure all sources and detector fibers on each participant’s head (helmet placement was rigorously performed using standard cranial landmarks such as nasion and inion). For each size, two different, but complementary, montage configurations were exploited, one per experimental session, and then combined to maximize coverage of the occipital and posterior temporoparietal cortices across sessions (see Fig. 1B). For each size, the order of montages was counterbalanced across participants and sessions, and combined with a further balancing procedure of the hand used to perform the behavioral task to prevent confounding effects. In each montage, sources and detectors were arranged to allow each detector to detect light from up to 16 time-multiplexed sources and to enable sources to emit light concurrently, avoiding cross-talks between channels. Depending on this time-multiplexing method, sources were sequentially switched on for 1.6 ms and switched off for 24 ms in each specific multiplexed set. This achieved a 25.6 ms lasting cycle and a sampling rate of 39.0625 Hz. Optical data were acquired from a total of 128 channels, even though only channels with source–detector distances ranging between 17.5 and 50 mm were considered. Longer or shorter distances were excluded because at longer distances optical signals may be unreliable, whereas shorter channels could measure light unable to reach the cerebral cortex. Moreover, a phase variability threshold of 200 ps was applied to identify channels affected by excessive noise or poor optical coupling. Importantly, before data collection, both montages were verified to comply with the predefined channel criteria, thereby reducing the risk of systematic variability due to montage differences.

Nineteen participants underwent structural MRI scans in a 1.5 Tesla Philips scanner at the Borgo Roma Hospital in Verona. A standard 15-channel head coil was employed, and 3D T1-weighted MR images were acquired with a magnetization-prepared rapid acquisition gradient echo (MPRAGE) sequence. Data acquisition parameters were as follows: phase encoding direction = anterior to posterior, voxel size = 0.5 × 0.5 × 1 mm, repetition time = 7.7 ms, echo time = 3.5 ms, field of view = 165 x 512 × 512 mm, flip angle = 8°.

For the remaining seven participants, structural MRI was not available, so an estimated MR-based head model was individually created using the Softaxic Optic system (SofTaxic, E.M.S., Bologna, Italy) combined with a 3D optical digitizer (Polaris Vicra, NDI, Waterloo, Canada). A warping procedure was employed based on four fiducial points (nasion, inion, and pre-auricular points) and a large number of scalp points corresponding to the holder positions of each helmet (247 and 259 points, respectively). Based on the scalp points digitization, a proper procedure generated a virtual reconstruction of the scalp surface. This reconstruction was then used to compute 345 scalp reference points based on the international 10–5 system (a set per participant) through which the averaged standard template MRIs were adjusted. Following each EROS session (during which helmet placement was systematically monitored), every source and detector holder location on the helmet, as well as fiducial points (nasion, inion, and pre-auricular points), were digitized for each participant. The digitized scalp locations were co-registered with the structural MR images or the estimated MRI using a specific procedure performed in the OCP software package (Optimized Co-registration Package, MATLAB code). The co-registration procedure (Chiarelli et al., 2015), essentially based on fiducial alignment processes (Whalen et al., 2008), was identical for both the MRI types. Finally, co-registered individual data were transformed to MNI space for the following analyses.

Optical data were collected by means of the ISS Corporation “Boxy” program and subsequently preprocessed using an in-house MATLAB-based software, namely P-POD (Pre-Processing of Optical Data). Data were corrected for phase wrapping, de-trended to remove drifts, and demeaned. Afterward, the time delay was obtained by converting the phase into picoseconds, adjusted to zero for each block. Later on, pulse artifacts were removed (heartbeat rate range 45–200) by using the heart pulse filter developed by Gratton and Corballis (1995), and data were band-pass filtered to remove frequencies outside the 0.5–15 Hz range. Specifically, the opacity value, defined as the product of the scattering and absorption coefficients, was calculated for each participant. Opacity values equal to zero or exceeding 3 standard deviations from the mean opacity across participants would have resulted in the participant’s exclusion from subsequent analyses. However, no participants met these criteria, therefore, none was excluded. Finally, output data were segmented into epochs time-locked either to the cue or the target onset and then averaged for each time point, channel, condition, and participant separately. The length of the epochs was thus the same for each EROS contrast (see below section *EROS analysis*), namely 1484 ms.

Statistical analyses of optical data were computed using the Opt-3d custom software package (Gratton, 2000). Mean optical signals were obtained by averaging those originating in channels whose diffusion paths converged in a given voxel (Wolf et al., 2014). Only channels providing a minimum of 20 trials each were included in the analyses. Phase delay data were baseline corrected using either a 200 ms pre-target interval or a 200 ms pre-cue interval (according to the analysis taken into consideration) and spatially smoothed with an 8-mm Gaussian kernel. Group-level *t*-statistics were calculated across participants and then converted to *z*-scores, for each voxel at each time point. *Z*-score maps were thus computed from the p-value for each t-test, subsequently undergoing the proper correction for multiple comparisons based on random field theory (Kiebel et al., 1999; Worsley et al., 1995). Eventually, according to the physical homogeneous model (Arridge & Schweiger, 1995; Gratton, 2000), *Z*-scores were weighted and orthogonally projected onto a template MNI brain’s coronal, sagittal, or axial surfaces.

The ROIs necessary in order to perform statistical analyses were identified by selecting those areas hypothesized to show attentional control modulation within areas included in EROS coverage (Fig. 1C). ROIs thus comprised occipital regions, that is, the primary visual area (V1) and the dorsal portion of the cuneus, dorso-parietal regions, that is, left and right SPLs (l/rSPL) and left and right IPSs (l/rIPS, representing the posterior portion of the DAN), and ventral regions, that is, left and right TPJs (l/rTPJ, representing the temporoparietal portion of the VAN). Specific ROIs coordinates were selected by matching anatomical coordinates of parietal, temporal, and occipital areas previously used in the literature (Baniqued et al., 2018) and the correspondent Brodmann areas incorporating these regions (i.e., BA17 for V1, BA18 and 19 for cuneus, BA7 for SPL, the intersection of BA7 and BA39 for IPS, BA39 for TPJ). Moreover, a potential overlapping of ROIs boundaries was eliminated by referring to the Bioimage Suite Web tool (https://bioimagesuiteweb.github.io/webapp/mni2tal.html). A 2-dimensional box-shaped structure described ROIs considered in this paper (the absence of the third dimension is due to the projection of the optical signal to the brain surface). Indeed, ROIs were examined availing of axial (x, y), sagittal (y, z), or coronal projections (x, z) only (see Table 1).

**Table 1.** MNI coordinates of selected ROIs.

Region	Projection	Coordinates			Involved BA
Right SPL	Axial	x = y =	0 -84	20 -64	7
Left SPL	Axial	x = y =	-20 -84	0 -64	7
Right IPS	Axial	x = y =	26 -87	40 -59	7–39
Left IPS	Axial	x = y =	-36 -87	-22 -59	7–39
Right TPJ	Sagittal	y = z =	-69 21	-49 41	39
V1	Coronal	x = z =	-10 -4	10 16	17
Cuneus	Coronal	x = z =	-10 20	10 40	18–19

### 2.5 Data analysis

#### 2.5.1 Behavioral data

Data were processed using MATLAB 2021b and analyzed with Jamovi for Windows, version 1.6.23. Reaction times (RTs) were evaluated to explore behavioral data. In each condition, anticipations (RTs < 150 ms) and responses deviating > 3SDs from the mean were excluded from the analyses. Mean RTs and the corresponding standard deviations (SDs) were measured for each of the four behavioral conditions (right target valid–invalid, left target valid–invalid) across participants, independently from target orientation (vertical or horizontal).

A repeated-measures analysis of variance (ANOVA) was then conducted on mean RTs, with *target side* (right/left) and *cue side* (right/left) as within-subject factors. Where needed, Bonferroni-corrected post hoc t-tests were applied.

#### 2.5.2 Functional data

##### 2.5.2.1 EROS analysis

The change in phase delay from baseline was the dependent variable for optical data analyses, averaged for each participant, condition, and time point. Specifically, one-tailed tests were performed on each ROI’s average at each latency. Statistical significance was measured by ROI peak Z scores with p < 0.05, adjusted for multiple comparisons (Kiebel et al., 1999; Worsley et al., 1995).

Concerning statistical analyses, trials were collapsed across target orientations, and three main contrasts were selected: all (valid and invalid) versus baseline, valid versus baseline, and invalid versus baseline.

For the first contrast (all versus baseline), all trials were collapsed together, baseline-corrected, and contrasted against it, namely the 200 ms time window preceding cue onset. To observe the orienting process after the cue onset, we analyzed latencies ranging between 0 and 307 ms (where 0 ms corresponds to the cue onset). Indeed, since cue occurrence typically triggers attentional deployment processes and our cue–target interval was variably randomized among trials (from 300 to 600 ms after the cue onset), in our pre-target analysis, we chose that specific time window considering that the latencies later than 300 ms after cue onset would not reveal homogeneous orienting responses because of the target presence in part of the trials.

For the other two contrasts (valid versus baseline and invalid versus baseline), valid and invalid trials were baseline corrected and separately contrasted against it (the 200 ms preceding the target onset), independently from the visual hemifield where the target occurred. Functional activity was evaluated from 0 to 650 ms (0 ms corresponds to the target onset).

##### 2.5.2.2 Granger causality analysis (GCA)

Forward GCA was calculated to characterize the directed functional interaction among activations in different regions at different time lags. The central idea underlying GCA is that directional influence from a specific region to another one, subsequent in time, can be deducted if past signal values of the first brain region support the prediction of that temporally later region’s present and future signal values. Therefore, identifying significant seeds within different ROIs is needed to perform GCA. A single seed consists of a time window whose predictive flow will be evaluated compared with another window of the same duration. In a nutshell, this approach investigates whether the activity of the seed ROI predicts the activity in the other ROIs at a later time at the individual level, thus providing the opportunity to highlight complex patterns across participants that conventional EROS analyses may not reveal. Statistical maps, which were obtained from the average of individual values calculated separately per ROI and contrast, were generated by means of the computation of *t statistics* and transformation into *z scores*. This procedure was conducted for each time lag. Subsequently, a correction for multiple comparisons within each ROI was performed using the same random field theory techniques (Kiebel et al., 1999; Worsley et al., 1995) used for EROS analysis. Directed functional interactions were studied at lags divided by 25.6 ms intervals (i.e., the sampling rate), proceeding from a lag of 0 ms until a lag of 358 ms, for a total of 15 time lags (which correspond to the same time points employed in EROS analyses). Statistically significant predicted ROI peaks were identified when z-scores exceeded the threshold of p < 0.05 at each specific lag. The timing associated with the significant lag, however, did not correspond to the actual activation of the predicted ROI. More precisely, as stated above, each of the 15 lags corresponded to a specific latency (expressed in ms), ranging from 0 ms for the first lag to 358 ms for the last lag. To determine the actual activation time, we defined a significant time window by adding the index of the significant lag (ranging from 1 to 15) to the starting seed interval. For example, if our starting seed was defined as a time window from 25 to 102 ms and the predicted significant lag was found at lag number 5, the resulting significant time window for that predicted ROI ranged from 127 to 204 ms (i.e., 25 ms +5 and 102 ms +5). Finally, within this significant time window, the timing of the most significant ROI peak was considered the actual activation time of the predicted ROI. The critical time window designated for each contrast started from the target onset onward for valid and invalid versus baseline contrasts. Instead, the time points corresponding to the 300 ms after cue onset were considered for the all versus baseline contrast. After focusing on lags in keeping with EROS results, exploratory analyses were run to examine additional predictive effects beyond the initial time window. These analyses followed the same statistical procedures and correction methods described above and were intended to provide a broader temporal perspective on inter-regional dynamics. Specifically, ROIs utilized as seeds corresponded to both ROIs exhibiting significant activations in EROS analyses and ROIs whose activity was predicted by previous in-time seeds.

## 3 Results

### 3.1 Behavioral results

**Fig. 2.**
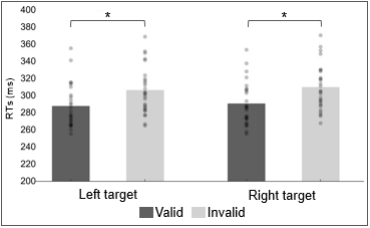
Behavioral results. Mean response times are plotted as a function of whether the target appeared in the right or left hemifield and as a function of whether the attention cue was valid or invalid. For each condition, individual data are plotted (gray dots) along with averaged values. Significant effects are marked with asterisks.

The ANOVA on mean RTs did not show a significant main effect of either the target side (F_(1,25)_ = 3.957, p < 0.058, ƞ^2^_p_ = 0.137) or the cue side (F_(1,25)_ = 0.611, p < 0.442, ƞ^2^_p_ = 0.024). In contrast, it revealed a significant interaction between the target side and the cue side (F_(1,25)_ = 67.460, p < 0.001, ƞ^2^_p_ = 0.730). Accordingly, Bonferroni-corrected post hoc t-tests indicated that mean RTs for right valid trials (288 ms) were statistically different compared with mean RTs for right invalid trials (307 ms, p < 0.001). Likewise, mean RTs for left valid trials (291 ms) were shown to be statistically different compared with mean RTs for left invalid trials (311 ms, p < 0.001). On the contrary, the RTs comparisons between left valid trials and right valid trials and between left invalid trials and right invalid trials did not reveal any statistically significant difference (see Fig. 2). These results suggest a reliable attentional orienting advantage in target detection: the valid condition always yields faster RTs; the target side factor did not modulate cue-related performances. Additionally, a post hoc power analysis (G*Power Version 3.1.9.4) based on the observed effect size of the interaction effect (ƞ²_p_ = 0.73; F = 1.64) was run, indicating an achieved power (1–β) > 0.99, confirming adequate sensitivity to detect these behavioral effects.

### 3.2 Functional results

#### 3.2.1 Orienting process-EROS results

**Fig. 3.**
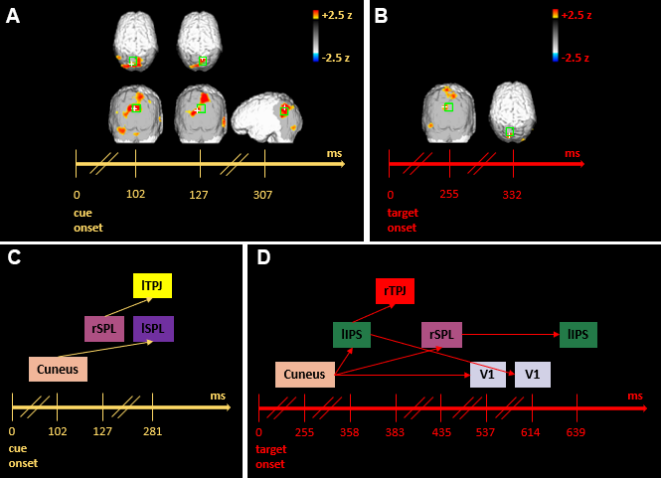
EROS orienting effects and Granger results. (A) EROS orienting effects after the cue onset. Significant statistical parametric maps of the z-score difference between all trials and baseline (corresponding to the 200 ms time window preceding the cue onset) are illustrated (activation threshold z-score = 2.0). Each map constitutes a 25.6 ms interval, within the 307 ms after the cue onset, in which significant effects occurred in selected ROIs (green boxes). The white cross shows the peak voxel within each ROI. (B) EROS orienting effects after the target onset. Significant statistical parametric maps of the z-score difference between valid trials and baseline (corresponding to the 200 ms preceding the target onset) are illustrated (activation threshold z-score = 2.0). Each map constitutes a 25.6 ms interval of 650 ms after the target onset, in which significant effects occurred in selected ROIs (green boxes). The white cross shows the peak voxel within each ROI. (C) GCA results of all versus baseline contrast. (D) GCA results of valid versus baseline contrast. For GCA, all dorsal and visual ROIs were chosen as seeds at different time lags. Here, each colored box corresponds to a specific ROI. Each arrow indicates a significant predictive link between the starting box/seed/ROI at a specific time lag and the matching box that depicts the predicted ROI at a subsequent time lag (see Table 2). The values indicated on the timeline refer to the peak activity for each ROI within the considered time interval.

Two EROS contrasts were carried out to explore the spatiotemporal dynamics underlying attentional orienting. The all versus baseline contrast (Fig. 3A, after cue onset) was conducted in the time window ranging from the cue onset to 300 ms post cue. In contrast, the valid versus baseline contrast (Fig. 3B, after target onset) considered the time window from target onset to 650 ms post-target.

Concerning the all versus baseline contrast, there was an increase of activation in both lSPL (*z* = 2.76; *z crit* = 2.68) and the dorsal portion of the cuneus (*z* = 3.27; *z crit* = 2.77) at 102 ms after the cue onset. The dorsal portion of the cuneus showed greater activity at 127 ms after the cue onset as well (*z* = 2.79; *z crit* = 2.63). At the same latency, we further observed greater activity occurring in rSPL (*z* = 2.75; *z crit* = 2.72). Subsequently, at a latency of 307 ms after the cue onset, a significant increase of activation was found in lTPJ (*z* = 2.94; *z crit* = 2.67) (see Fig. 3A).

Comparing valid trials with the baseline, we found a significant increase of activation in the dorsal portion of the cuneus at 255 ms (*z* = 2.90; *z crit* = 2.74) after target onset. Moreover, significant activity was found in lSPL activation at a latency of 332 ms after target onset (*z* = 2.45; *z crit* = 2.39). Importantly, the functional results from both contrasts corroborate the involvement of both bilateral dorso-parietal and visual areas in attentional orienting (see Fig. 3B). See Supplementary Figure S1 A and B for the time traces of the mean activation of each significant ROI.

#### 3.2.2 Orienting process-GCA results

GCA applied to the all versus baseline contrast allowed us to explore the stream of predictive interfaces straight after cue occurrence. In particular, the significant EROS activity in cuneus, peaking at 102 ms after the cue onset, was predictive of activity in lSPL corresponding to a peak at 307 ms. Moreover, peak activation of 127 ms in rSPL predicted peak activation in lTPJ at 307 ms after the cue onset (see Table 2 for significant lags and the corresponding time windows). As a result, a predictive dialogue among visual and dorsal areas emerged along with a predictive relationship between dorsal and ventral regions (see Fig. 3C and Table 2 for significant lags, the corresponding time windows, and statistics).

**Table 2.** Granger analyses—orienting results.

Seed ROI	Seed interval (ms)	Peak activity (ms)	Predicted ROI(s) (PR)	PR interval (ms)	PR peak activity (ms)	Sig. Lag	Statistics
***After cue onset***							
Cuneus	0–204	102	lSPL	204–435	307	204	*z* = 3.35, *z crit* = 2.94
rSPL	51–204	127	lTPJ	25–307	307	76	*z* = 2.83, *z crit* = 2.71
***After target onset***							
Cuneus	153–307	255	lIPS	281–435	358	127	*z* = 3.01, *z crit* = 2.97
			rSPL V1	358–511 386–537	435 537	204 230	*z* = 3.40, *z crit* = 3.00 *z* = 3.18, *z crit* = 2.90
lIPS	281–435	358	rTPJ	281–384	383	332	*z* = 3.65, *z crit* = 2.88
			V1	409–537	614	25	*z* = 2.97, *z crit* = 2.85
rSPL	358–486	435	lIPS	639–767	639	281	*z* = 3.22, *z crit* = 3.04

By applying GCA to the valid versus baseline contrast, we could investigate predictive relationships between our ROIs in the time window after target onset. The stream of predictive influences began from activity in cuneus, which was predictive of activity in lIPS (peak activation 358 ms), rSPL (peak activation 435 ms), and V1 (peak activation 537 ms). The former predicted ROI (i.e., lIPS peaking at 358 ms) was, in turn, predictive of activity in rTPJ (peak activation 383 ms) and V1 (peak activation 614 ms), while rSPL predicted orienting activity in lIPS at a later lag inferring peak at 639 ms (see Fig. 3D and Table 2 for significant lags, the corresponding time windows, and statistics).

GCA applied to the valid versus baseline contrast displays results analogous to those of the all versus baseline contrast, indicating a bilateral dorsal–parietal and visual areas engagement, mainly characterized by bi-directional predictive connections between dorsal and visual area activations.

The prediction of rTPJ from a seed detected in lIPS, in the valid versus baseline contrast, appears to be an uncommon outcome. RTPJ does not seem to have a specific role in this process, mainly because it, in turn, does not predict any other subsequent significant activation, thus revealing mere functional connectivity between these areas but not effective connectivity, that is, related to the task at hand.

#### 3.2.3 Reorienting process-EROS results

Invalid trials were contrasted with the baseline to unveil the neural dynamics responsible for attentional reorienting. The analyzed time window is the same as the valid versus baseline contrast.

**Fig. 4.**
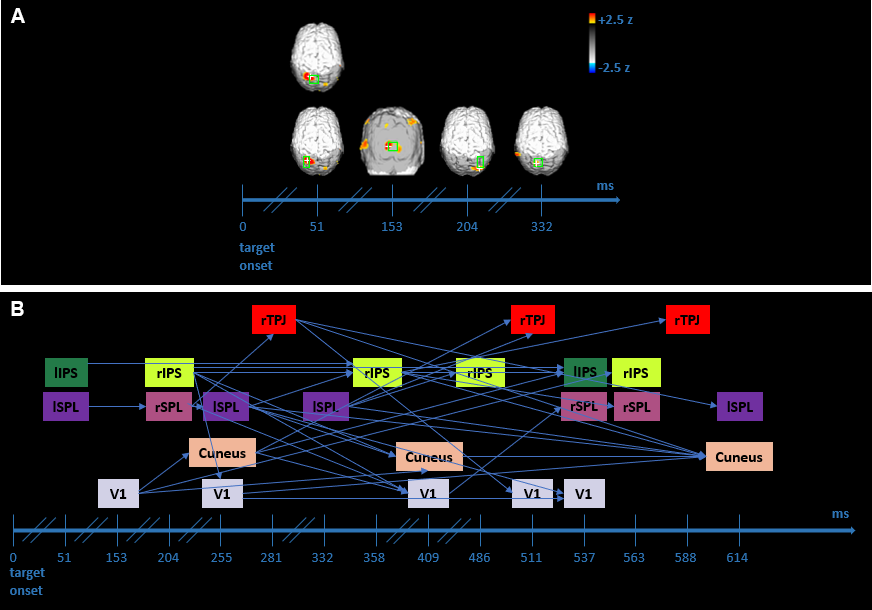
Reorienting EROS effects and Granger results. (A) EROS reorienting effects after the target onset. Significant statistical parametric maps of the z-score difference between invalid trials and baseline (corresponding to the 200 ms preceding the target onset) are illustrated (activation threshold z-score = 2.0). Each map constitutes a 25.6 ms interval of 650 ms after target onset, in which significant effects occurred in selected ROIs (green boxes). The peak voxel is shown by the white cross within each ROI. (B) GCA results of invalid versus baseline contrast. Again, each arrow indicates a significant predictive link between the starting box/seed/ROI at a precise time lag, and the matching box that depicts the predicted ROI at a subsequent time lag (see Table 3). The values reported on the timeline refer to the peak activity within the considered time interval for each ROI.

We observed greater activity in both lIPS (*z* = 3.60; *z crit* = 2.76) and lSPL (*z* = 3.26; *z crit* = 2.92) at 51 ms after target onset. Then, we found stronger activation in V1 (*z* = 3.2; *z crit* = 2.47) at 153 ms and in rIPS at 204 ms (*z* = 2.49; *z crit* = 2.46). Finally, greater activity was observed in lSPL (*z* = 2.623; *z crit* = 2.62) at 332 ms after target onset (see Fig. 4A). The present reorienting results seem to reveal similar dynamics compared with attentional orienting: a robust engagement of the dorso-parietal network along with visual areas. It should be noted that these findings do not indicate the recruitment of the ventral network, in particular of rTPJ, in this type of process.

Additionally, a post hoc power analysis (G*Power Version 3.1.9.4) was conducted on the mean activation (across the three contrasts) observed in a common region, that is, lSPL, (Cohen’s d = 0.579, α = 0.05, one-tailed hypothesis) obtaining an achieved power (1-β) of 0.889 and confirming the statistical sensitivity of our main neural effects. See Supplementary Figure S1 C for the time traces of the mean activation of each significant ROI.

#### 3.2.4 Reorienting process-GCA results

We availed of GCA to better understand the role of the ventral network, especially of rTPJ, and its predictive relationships with dorsal and visual areas in reorienting operations. Seeds identified earlier in time revealed predictive connections so that dorsal areas predicted activity in dorsal areas only (i.e., lSPL, peaking at 51 ms, is predictive of activity in rSPL peaking at 204 ms; lIPS, peaking at 51 ms, is predictive of activity in rIPS peaking at 358 ms), and, similarly, visual areas predicted activity in visual ROIs exclusively (i.e., V1, peaking at 153 ms, is predictive of activity in cuneus peaking at 255 and 409 ms). Subsequently, the sustained recurrent reciprocal prediction pattern between dorsal and visual areas turned out. Indeed, activity in dorsal areas was predictive of activity in dorsal and visual areas, and activity in visual areas was predictive of activity in visual and dorsal ROIs. For instance, a seed identified in rIPS at a range between 102 and 255 ms (peak activation 204 ms) predicted activity in rIPS (peak activation 358 ms) and visual areas, including V1 (peak activation both 255 and 409 ms) and cuneus (peak activation 409 ms).

Moreover, activity in cuneus corresponding to a peak at 255 (which was previously predicted by V1 peaking at 153 ms) resulted in predicting activity firstly in V1 (peak activation 409 ms) and secondly in lIPS (peak activation 537 ms). Significantly, rTPJ was also involved in these attentional predictive processes. Activity in rTPJ was predicted by dorsal and visual areas at different and belated time lags. More specifically, activity in rSPL (peak activation 204) was predictive of activity in rTPJ with a peak activation at 281 ms. Furthermore, a seed identified in cuneus (peak activation 255) predicted activity in rTPJ at two later lags, inferring peaks at 511 and 588 ms (see Fig. 4B and Table 3 for significant lags, the corresponding time windows, and statistics). The main difference concerning orienting results is that in the context of attentional reorienting, rTPJ predicts, in turn, activity in visual and dorsal ROIs at subsequent lags, while in the orienting process, it does not carry on the stream of predictive influences.

**Table 3.** Granger analyses—reorienting results.

Seed ROI	Seed interval (ms)	Peak activity (ms)	Predicted ROI(s) (PR)	PR interval (ms)	PR peak activity (ms)	Sig. Lag	Statistics
lIPS	0–127	51	rIPS	255–383	409	255	*z* = 3.46, *z crit* = 2.91
lSPL	0–127	51	rSPL	179–307	307	179	*z* = 3.13, *z crit* = 3.05
V1	76–230	153	Cuneus	153–307	255	76	*z* = 2.96, *z crit* = 2.85
			Cuneus	358–511	409	281	*z* = 2.94, *z crit* = 2.87
			rIPS	435–588	563	358	*z* = 2.87, *z crit* = 2.81
rIPS	102–255	204	V1	332–511	255	153	*z* = 3.28, *z crit* = 2.99
			V1	307–486	409	179	*z* = 3.08, *z crit* = 2.90
			Cuneus	409–537	409	179	*z* = 3.01, *z crit* = 2.84
			rIPS	332–486	358	230	*z* = 3.28, *z crit* = 2.99
rSPL	76–255	204	lSPL	230–409	255	153	*z* = 3.01, *z crit* = 2.98
			rTPJ	127–307	281	51	*z* = 3.35, *z crit* = 2.75
			rIPS	230–409	358	153	*z* = 2.75, *z crit* = 2.72
			V1	307–486	409	204	*z* = 3.04, *z crit* = 2.89
			lIPS	435–614	537	358	*z* = 2.98, *z crit* = 2.93
lSPL	204–307	255	rIPS	332–435	358	127	*z* = 3.09, *z crit* = 3.02
			V1	486–588	537	281	*z* = 4.12, *z crit* = 3.08
			Cuneus	563–665	614	358	*z* = 3.28, *z crit* = 3.04
V1	179–332	255	V1	511–665	537	332	*z* = 3.20, *z crit* = 3.15
			Cuneus	460–614	614	281	*z* = 3.13, *z crit* = 2.58
Cuneus	179–358	255	V1	307–435	409	127	*z* = 3.24, *z crit* = 2.81
			rTPJ	460–639	511	281	*z* = 3.01, *z crit* = 2.89
			lIPS	486–665	537	307	*z* = 3.15, *z crit* = 2.56
rTPJ	230–383	281	V1	358–511	511	127	*z* = 3.09, *z crit* = 2.84
			Cuneus	588–742	61	358	*z* = 2.91, *z crit* = 2.87
			lSPL	511–665	614/639	281	*z* = 2.91, *z crit* = 2.84
lSPL	281–383	332	Cuneus	307–409	409	25	*z* = 3.04, *z crit* = 3.03
			rIPS	409–511	486	127	*z* = 3.04, *z crit* = 2.98
			rTPJ	409–511	511	127	*z* = 3.56, *z crit* = 3.19
			Cuneus	486–639	614	204	*z* = 3.34, *z crit* = 3.20
rIPS	281–435	358	lIPS	537–691	537	255	*z* = 3.40, *z crit* = 2.89
			rSPL	563–716	563	358	*z* = 3.24, *z crit* = 2.95
			rTPJ	588–742	588	307	*z* = 3.17, *z crit* = 2.95
			Cuneus	563–716	614	281	*z* = 2.97, *z crit* = 2.96
V1	332–486	409	rSPL	409–563	537	76	*z* = 3.25, *z crit* = 3.05
cuneus	307–537	409	Cuneus	511–742	614	204	*z* = 3.23, *z crit* = 3.10

Overall, these reorienting findings underline a predictive model whereby dorsal and visual areas predict themselves at an early stage. Afterward, a similar prediction pattern to that revealed in orienting GCA develops, showing a mutually predictive interface between dorsal and visual areas. Finally, the ventral network, corresponding to rTPJ, comes into play by reciprocally predicting both dorsal and visual regions.

## 4 Discussion

The purpose of the present study was to reveal the neural substrates of visuospatial attentional processes from both a spatial and temporal point of view by coupling a spatial cueing paradigm with fast optical imaging data. This paradigm is usually characterized by the processing of valid and invalid trials. Valid trials typically engender a voluntary deployment of attention (after interpreting the predictive cue) and a cue-related orienting response, entailing distinct subprocesses: disengaging attention from the central fixation cross and shifting and engaging attention to the cued location. A mismatch between the cued and actual target location is instead triggered by invalid trials, requiring further attentional mechanisms, that is, disengaging, shifting, and re-engaging attention to the correct location (Natale et al., 2009). By availing of the EROS technique, we intended to disclose the brain regions responsible for orienting and reorienting processes. By integrating a good temporal and spatial localization ability, EROS enabled us to identify the timing of our ROIs’ activation over the two attentional mechanisms (Colombari et al., 2024; X. Xiao et al., 2020). Furthermore, GCA unveiled the predictive and mutual interactions among the different ROIs, whose exact nature is still unclear but crucial to thoroughly understanding visuospatial attentional dynamics.

We explored the ability to intentionally orient attention to a spatially cued, lateralized visual stimuli by considering two EROS contrasts: all trials (regardless of the cue validity) versus baseline and valid trials versus baseline. The former contrast was performed to investigate the spatiotemporal correlates related to anticipatory visual orienting taking place across the cue–target interval. We instead compared valid trials with the baseline to unveil the brain regions and their timings of activation subserving deployment and maintenance of visuospatial attention after the target onset. Our findings show an overarching involvement of dorso-parietal and visual areas in voluntary orienting of attention, confirming the role played by the DAN and occipital regions in managing this process (Ptak & Schnider, 2010): a bilateral SPL and an extra-striate engagement have been highlighted both during the cue–target interval and after the target onset. Our cue-related results agree with previous studies employing spatial cueing paradigms. Mayrhofer and colleagues (Mayrhofer et al., 2019) explored anticipatory pre-target activity linked to the informative cue, suggesting a correlation between activations in brain regions overlapping with our bilateral SPL ROIs and selective attentional behavioral effects on task performance. In addition, Vandenberghe et al. (2012) revised structural lesion studies investigating the role of the superior parietal cortex in spatial attentional disorders which supported an involvement of SPL in cue-related attentional shifting independently from the cued direction. Concerning extra-striate areas, our EROS results reveal a solid activation of cuneus, whose contribution is also supported by our GCA performed on after-cue-onset data. Indeed, cuneus has been found to predict lSPL activity at a different time lag, pointing out a dorso-visual predictive interaction underlying the intentional deployment of visuospatial attention after the presentation of an informative cue. This finding is in line with prior evidence indicating that early activations of extra-striate cortices account for endogenously orienting and the contribution of SPL in triggering a shift of attention, especially when it is decoupled from central fixation (Kelley et al., 2008; Rihs et al., 2009). The critical involvement of cuneus and SPL has been uncovered after target onset as well. These findings are totally in accordance with the results highlighted in Parisi et al. (2020), where cuneus and SPL were the main outcomes of EROS after-target-onset analyses obtained from a discrimination spatial cueing task. As to GCA results, they seem to bring out a dorso-visual mutually predictive interplay also over this time window, further supporting the crucial role of dorso-parietal nodes of the DAN (i.e., SPL) and extra-striate regions (i.e., cuneus) in a top–down attentional information flow (Doesburg et al., 2016; Proskovec et al., 2018). Moreover, our GCA results are in line with studies unfolding the implication of IPS and early visual regions during sustained attention, showing the coming into play of V1 and IPS in a dorso-visual reciprocally predictive interface. Indeed, these regions, especially IPS, have been revealed to be implicated in top–down control attentional mechanisms by representing sustained states of peripheral attention (Kelley et al., 2008; Parisi et al., 2020).

Our orienting analyses also exhibit seemingly uncommon findings, that is, the involvement of the VAN in covertly orienting visuospatial attention. Specifically, lTPJ and rTPJ have been shown to be engaged in performing an attentional orienting process after the cue onset and after the target onset, respectively. Nevertheless, concerning the contribution of lTPJ, recent fMRI studies support this evidence by highlighting lTPJ activations in spatial attentional tasks (Abrahamse & Silvetti, 2016; Doricchi et al., 2010; Wisniewski et al., 2015). DiQuattro and Geng (2011), by availing of a visual search paradigm, demonstrated the role of lTPJ in detecting informative salient aspects and using them to initiate endogenous and effective attentional orienting. Moreover, a left-hemispheric relevance in managing different aspects of attentional mechanisms has been highlighted in broadly similar research (Dragone et al., 2015; Orlandi & Proverbio, 2019; Silvetti et al., 2016). Hence, we believe that it would be of great interest to deepen the left-hemispheric contribution by means of additional studies.

More infrequent could appear the directed influence observed from lIPS to rTPJ after the target onset, which represents a brain region typically active during reorienting processes. We believe that this result could not have a specific contribution to the orienting process, given that it emerges in GCA only, and does not predict, in turn, any other ROIs, thus likely not contributing to carrying on the attentional stream. However, a TMS-fMRI study by Leitão et al. (2015) administering a sustained spatial attention paradigm, which did not include reorienting mechanisms, highlighted the importance of IPS for modulating neural processes in the rTPJ. Further research is, thus, needed to ascertain a possible contribution of rTPJ in orienting processes.

To summarize, our results suggest a predictive pattern between dorsal and visual regions that persists in both the analyzed functional contrasts, lasting over the whole orienting process, from the cue occurrence until after the target onset. This dorsal and visual network, whose main components are bilateral SPL and the dorsal portion of the cuneus, is responsible for each neural step of endogenous attentional orienting. Moreover, our findings exhibit new evidence of the engagement of the bilateral ventral network in this cognitive mechanism.

In the present work, the contrast between invalid trials and the baseline was also considered with the purpose of examining visuospatial attentional reorienting. Indeed, comparing invalid trials with the baseline allowed us to segregate the brain regions, and their timing of activation, activated by targets occurring at an uncued location after an endogenous attentional expectation generated by the cue. Our results point out a clear implication of bilateral dorso-parietal regions along with visual areas in responding to invalid trials considering an after-target onset time window. This evidence is in keeping with previous studies supporting the involvement of the DAN in reorienting attention toward unexpected target locations (Doricchi et al., 2010; Vossel et al., 2009, 2012). More precisely, we observed bilateral recruitment of IPS while the importance of lSPL emerged again. The stated above cognitive stages (i.e., perceiving a mismatch between expectations and reality, disengaging, shifting, and re-engaging attention from the cued location to the correct one) appear underpinned by the aforementioned dorso-parietal regions differently, as suggested by Spadone and colleagues (Spadone et al., 2021): SPL was found to be entailed by shifting and reorienting attention processes, while IPS was entailed by sustaining attention processes.

This last evidence seems to be in line with our current EROS results, but to better understand how these brain regions interact to accomplish their roles, we further analyzed data by applying GCA. Surprisingly, our invalid versus baseline contrast did not bring out any significant contribution of the ventral network, in particular of rTPJ, in endogenous reorienting processes. Again, GCA helped us expand our functional results and better comprehend the predictive relationships among dorso-parietal, ventral, and visual areas during reorientation of attention after encoding the discrepancy between cue-related expectancy and the actual event.

At first general sight, predictive connections are several more than those involved in the orienting process. In the very beginning, we noticed an early predictive interface among the same cortical regions (i.e., dorso-parietal areas predicted dorso-parietal areas and visual areas predicted visual areas), followed by a dorso-visual stream in which dorso-parietal (bilateral SPL and bilateral IPS) and visual areas (V1 and cuneus) reciprocally predict each other in a post-target time window ranging between 200 and 600 ms. In addition, these data show the coming into play of the ventral network, embodied by rTPJ. The activity of rTPJ is actually influenced by both dorso-parietal (i.e., rSPL and rIPS) and visual regions (i.e., cuneus) at later timeframes after the target onset, highlighting a recurrent predictive pattern. Indeed, rTPJ exerts, in turn, predictive effects on both visual and dorso-parietal regions, subsequently in time.

Overall, our reorienting results are partially in accordance with the already cited MEG study by Proskovec et al. (2018), investigating attentional reorienting in a visuospatial Posner-like task. The authors found greater functional connectivity in the alpha band across bilateral SPL, highlighting a significant contribution of this region in serving control-related attentional processes. Similarly, we reported in EROS and GCA results, respectively, stronger activations and predictive relationships between bilateral SPL, which is thus fundamental in controlling and shifting attention during invalid trials. However, Proskovec et al. (2018) suggested the disengagement of attention from the cued incorrect location to be managed by the VAN, more precisely by the right IFG, which revealed increased theta activity during early processing of invalid targets. This interpretation does not fit with our results, which, instead, seem to indicate that disengaging attention from the invalidly cued location and shifting and re-engaging it to the uncued location are subserved by a persistent and mutually predictive dialogue among dorso-parietal and visual regions. Furthermore, rTPJ, which in our study is the primary representing node of the VAN, seems not to participate in this triggering and reorienting process by being predicted later in time and unveiling the timing of activations typical of P3b (300–500 ms post-target). This evidence enables us, on one side, to discard the idea of the VAN as a “circuit-breaker” or a trigger of the reorienting process and, on the other side, to support the “Contextual Updating” hypothesis (Geng & Vossel, 2013; Polich, 2007), sustaining a post-perceptual and supervision connotation of the role of rTPJ. This supervision should be fulfilled by constantly updating internal models of the behavioral context (Doricchi et al., 2022). More precisely, rTPJ should be responsible for updating the probabilistic cue–target contingencies in a trial-by-trial manner to preserve or change the attentional task set (Doricchi et al., 2010). This monitoring function seems to be more relevant when invalid trials occur. Indeed, due to their unexpected nature, they would generate a stronger need to update the internal model about the cue–target association to perform accurately in subsequent trials. Nevertheless, it has been demonstrated that valid trials also yield a post-perceptual updating mechanism (Arjona Valladares et al., 2017). This evidence would be in accordance with our GCA-orienting findings, highlighting the prediction of activity in rTPJ carried out by dorso-parietal regions. We can speculate that the difference between valid and invalid trials in the strength of engendering an updating process could be supported by our results: rTPJ activity predicted during valid trials does not further continue the flow of information, while rTPJ activity predicted during invalid trials exerts in turn directed influences on, later in time, DAN, and visual regions, unfolding a strengthened procedure of integrating novel information with the preexisting internal model.

With respect to the absence of significant activity of rTPJ in invalid versus baseline EROS contrast, we believe this could be due to the specific paradigm we employed. Our location-cueing paradigm was purposely implemented to investigate the spatiotemporal dynamics of orienting and reorienting processes requiring a low cognitive demand. At a behavioral level, these neural mechanisms are embodied by both the validity effect and the contextual updating effect. The validity effect results from the advantage of being attentionally oriented to the cued location and the cost produced by redirecting attentional resources from the location indicated by the cue to the uncued one. It is a general, very strong effect, globally and uniformly distributed over the whole task performance, mainly representing the reorienting process. Therefore, its robustness and global nature likely prevented it from being influenced by the current low cognitive load request. In contrast, the contextual updating process consists of a cognitive assessment concerning the validity/invalidity of the current trial, causing behavioral effects that are transferred to the subsequent trial. This effect, which seems to be subserved by rTPJ, is more particularly distributed than the validity effect, and more dependent on specific manipulations of the employed paradigm, which could thus had been impacted by the low cognitive load request. Consequently, we supposed the updating process has been prevented from being detected by EROS analyses, probably overwhelmed by the stronger and more overarching validity effect. This apparent issue has been easily overcome by applying GCA to EROS functional data, letting us observe the neural behavior of rTPJ during visuospatial attentional events with a high level of reliability.

Despite the novelty and the scientific contribution of this study, it was not without limitations. The main one was the impossibility of covering frontal areas by means of our EROS montages, preventing us from investigating the activity of FEF and VFC, which are known to have a fundamental involvement in visuospatial attentional processes (Proskovec et al., 2018; Spadone et al., 2021; Vossel et al., 2012). These regions are involved in both orienting and reorienting processes. More specifically, FEF interacts with IPS in modulating the visual cortex in a top–down manner during attentional orienting (Bressler et al., 2008; Vossel et al., 2012). However, the precise direction of this interaction has not been established yet. Then, a significant contribution of the right MFG and right IFG (both parts of the VFC) was revealed, linked to invalid trials selectively (Vossel et al., 2006). Actually, spontaneous activity was found in the right MFG to be associated with both dorsal and ventral areas, suggesting its crucial involvement in both attentional systems (Fox et al., 2006). Therefore, further EROS studies should try to include frontal regions in functional analyses to examine how they take part in the predictive visual, dorso-parietal, and ventral relationships engaged by endogenous attention.

## 5 Conclusions

To conclude, the present study intended to disclose the functional interplays among the cortical areas corresponding to the posterior nodes of the DAN and the VAN, underlying covert endogenous orienting and reorienting processes evoked through a detection Posner-like paradigm. Taken together, our findings are in keeping with Parisi et al. (2020), supporting the role of a dorso-visual network in controlling, directing, and re-directing visuospatial attention in both orienting and reorienting mechanisms. Regarding the contribution of rTPJ, both studies suggest a post-perceptual role in updating the internal model of the cue–target relationship as a function of new information on a trial-by-trial basis. Finally, the current study discloses a quite robust implication of the lTPJ in the cue–target orienting procedure. Therefore, our evidence confirms and expands the current literature by demonstrating the likely neural underpinnings of top–down control and updating attentional mechanisms.

## Supplementary Material

Supplementary Material

## Data Availability

The behavioral dataset and the pre-processed EROS data are openly available in the OSF repository at https://doi.org/10.17605/OSF.IO/BG9XP. Raw EROS data are available from the corresponding author on reasonable request, as no public online repository has sufficient capacity to store them freely.
