# Supplementary material for: Neural dynamics of visuospatial endogenous attention: Event-related optical signal evidence from posterior brain areas
Source: Imaging Neurosci (Camb). 2026 Mar 23;4:IMAG.a.1176. doi: 10.1162/IMAG.a.1176 (PMC13010356; doi:10.1162/IMAG.a.1176)
Supplement: Supplementary Material [file IMAG.a.1176_supp.pdf]

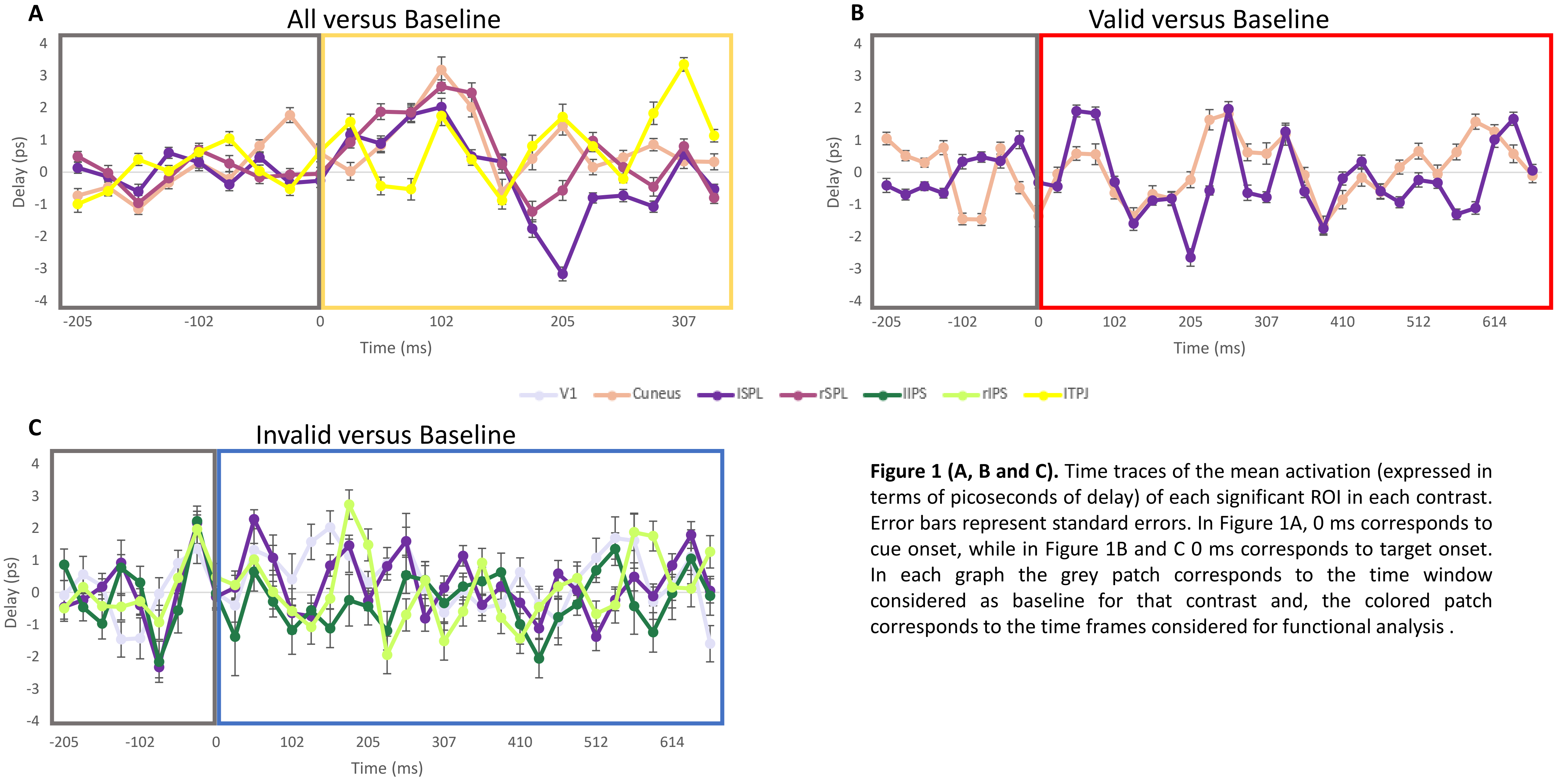

Figure 1 (A, B and C). Time traces of the mean activation (expressed in terms of picoseconds of delay) of each significant ROI in each contrast. Error bars represent standard errors. In Figure 1A, 0 ms corresponds to cue onset, while in Figure 1B and C 0 ms corresponds to target onset. In each graph the grey patch corresponds to the time window considered as baseline for that contrast and, the colored patch corresponds to the time frames considered for functional analysis .
